# Subclinical hypothyroidism predicts outcome in heart failure: insights from the T.O.S.CA. registry

**DOI:** 10.1007/s11739-024-03665-w

**Published:** 2024-06-08

**Authors:** Mariarosaria De Luca, Roberta D’Assante, Massimo Iacoviello, Vincenzo Triggiani, Giuseppe Rengo, Alfredo De Giorgi, Giuseppe Limongelli, Daniele Masarone, Maurizio Volterrani, Antonio Mancini, Andrea Passantino, Pasquale Perrone Filardi, Angela Sciacqua, Olga Vriz, Roberto Castello, Michela Campo, Giuseppe Lisco, Pietro Amedeo Modesti, Stefania Paolillo, Toru Suzuki, Andrea Salzano, Alberto Maria Marra, Eduardo Bossone, Antonio Cittadini, T.O.S.CA. Investigators

**Affiliations:** 1 Department of Translational Medical Sciences, Federico II University, Naples, Italy; 2 Cardiology Unit, Department of Medical and Surgical Sciences, University of Foggia, 71122 Foggia, Italy; 3 Interdisciplinary Department of Medicine-Section of Internal Medicine, Geriatrics, Endocrinology and Rare Diseases, University of Bari ‘A Moro’, Bari, Italy; 4 Istituti Clinici Scientifici ICS Maugeri-S.P.A.-Istituti Di Ricovero E Cura a Carattere Scientifico (IRCCS) Istituto Scientifico Di Telese Terme, Telese, Italy; 5 Clinical Medicine Unit, Department of Medicine, Azienda Ospedaliero-Universitaria S. Anna, Ferrara, Italy; 6 Division of Cardiology, Monaldi Hospital, Azienda Ospedaliera Dei Colli, University of Campania Luigi Vanvitelli, Caserta, Italy; 7 Department of Medical Sciences, IRCCS San Raffaele Pisana, Rome, Italy; 8 Operative Unit of Endocrinology, Catholic University of the Sacred Heart, Rome, Italy; 9 Scientific Clinical Institutes Maugeri, IRCCS, Bari, Italy; 10 Department of Advanced Biomedical Sciences, Federico II University, Naples, Italy; 11 Department of Medical and Surgical Sciences, University Magna Græcia of Catanzaro, Catanzaro, Italy; 12 Heart Center Department, King Faisal Hospital & Research Center, Riyadh, Saudi Arabia; 13 Division of General Medicine, Azienda Ospedaliera Universitaria Integrata, Verona, Italy; 14 Department of Medical and Surgical Sciences, Unit of Endocrinology and Metabolic Diseases, University of Foggia, Foggia, Italy; 15 Department of Medical Sciences, School of Medicine, Pharmacy and Prevention, University of Ferrara, Ferrara, Italy; 16 Department of Cardiovascular Sciences, University of Leicester, NIHR Biomedical Research Centre, Glenfield Hospital, Leicester, UK; 17 Cardiology Unit, A.O.R.N. Antonio Cardarelli, Naples, Italy; 18 Department of Public Health, University “Federico II” of Naples, Naples, Italy; 19 Division of Internal Medicine & Metabolism & Rehabilitation, University Federico II, 80131 Naples, Italy

**Keywords:** Heart failure, Subclinical hypothyroidism, Mortality, Hormones

## Abstract

Subclinical hypothyroidism (SH), defined as increased serum thyroid-stimulating hormone (TSH) with normal free T4 (fT4) levels, is frequently observed in the general population. Prevalence ranges from 0.6% to 1.8% in the adult population, depending on age, sex, and iodine intake. Several studies reported a worse prognosis in patients with heart failure with reduced ejection fraction (HFrEF) and SH, but they considered heterogeneous populations suffering mainly from severe SH. Aim of this study was to evaluate if SH was independently associated with the occurrence of cardiovascular death considering 30 months of follow-up. 277 HFrEF patients enrolled in the prospective, multicenter, observational T.O.S.CA. (Terapia Ormonale Scompenso CArdiaco) registry, were included in this analysis. Patients were divided into two groups according to the presence of SH (serum TSH levels > 4.5 mIU/L with normal fT4 levels). Data regarding clinical status, echocardiography, and survival were analyzed. Twenty-three patients displayed SH (87% mild vs 13% severe), while 254 were euthyroid. No differences were found in terms of age, sex, HF etiology, and left ventricular ejection fraction. When compared with the euthyroid group, SH patients showed higher TSH levels (7.7 ± 4.1 vs 1.6 ± 0.9, *p* < 0.001), as expected, with comparable levels of fT4 (1.3 ± 0.3 vs 1.3 ± 0.3, *p* = NS). When corrected for established predictors of poor outcome in HF, the presence of SH resulted to be an independent predictor of cardiovascular mortality (HR: 2.96; 5–95% CI:1.13–7.74; *p* = 0.03). Since thyroid tests are widely available and inexpensive, they should be performed in HF patients to detect subclinical disorders, evaluate replacement therapy, and improve prognosis.

## Introduction

Chronic heart failure (CHF) is a major and growing healthcare issue with increasing prevalence worldwide, high medical costs and burdened by a still unacceptable 5-year mortality rate close to 50% [1, 2]. Despite advances due to pharmacological and non-pharmacological therapies, patients with HF often display concomitant comorbidities that elevate their level of complexity, increase patients’ frailty and, most importantly, worsen their already poor prognosis [3]. Complementing this assumption, efforts have been made in recent years in identifying clusters of patients with aggressive comorbidities who may nevertheless benefit from innovative and promising therapeutic approaches. Thyroid dysfunctions represent one of the most investigated hormonal axes in HF patients [4] and has been associated with clinically relevant outcomes, including increased cardiac-related hospitalization and mortality [5, 6]. Current guidelines recommend assessment of thyroid function in all HF patients as both hypo- and hyperthyroidism may cause or precipitate HF [7]. Additionally, new evidence suggests that subclinical hypothyroidism (SH) is linked to cardiovascular diseases and HF, as it impairs lipid profile, endothelial function, blood pressure values, coagulative homeostasis and promotes myocardial remodeling [8–10]. However, to date the impact of SH in the prognosis of outpatients with CHF remains poorly investigated. Moreover, most investigations support implementation of replacement therapy in this subset of patients. In this scenario, the aim of the present study was to evaluate the prognostic impact of SH in a population of well-characterized CHF patients enrolled in the T.O.S.CA. registry, a multicenter, prospective observational registry focused on hormonal abnormalities in HF [11].

## Methods

### Study population and study procedures

A total of 277 patients with HF with reduced ejection fraction (HFrEF) enrolled within the prospective, multicentric, observational T.O.S.CA. (Terapia Ormonale Scompenso CArdiaco) registry were included in this analysis. Study design has been previously described [11]. Briefly, the T.O.S.CA. registry enrolled 480 consecutive stable CHF patients with left ventricular ejection fraction (LVEF) ≤ 45%, on stable medications for at least 3 months before enrollment, including any beta-blocker which had to be started at least 6 months before entering the study. Exclusion criteria included recent acute decompensation or acute coronary syndrome, severe liver cirrhosis in Child–Turcotte–Pugh stage B or C, clinically relevant kidney disease (creatinine level > 2.5 mg/dl), active malignancy and current hormonal treatment or overt endocrine diseases. In this sub-analysis, we excluded patients with altered fT4, subclinical hyperthyroidism, and low T3 syndrome. A total of 277 patients were selected and divided according to the presence of SH. SH was defined as TSH levels higher than 4.5 mIU/L with normal fT4 levels and classified according to the elevation in serum TSH level: mildly increased TSH levels (4.0–10.0 mU/l), and more severely increased TSH value(> 10 mU/l) [12]. Patient demographics, blood chemistry measurements, and clinical characteristics were recorded at the time of the enrollment. Echocardiography (including two-dimensional, Doppler, Color, and tissue Doppler analysis) was also performed. Primary endpoint of the study was cardiovascular death.

The study protocol was approved by the Ethics Committees of all participating centers and all patients gave written informed consent. All procedures were in accordance with the ethical standards of the institutional and/or national research committee and with the 1964 Helsinki declaration and its later amendments or comparable ethical standards.

### Statistical analysis

Normally distributed continuous variables were expressed as mean ± standard deviation. Categorical variables were expressed as counts and percentages. Patients were compared according to the presence of SH or normal TSH levels with unpaired Student’s *t*-test. Categorical variables were evaluated with *χ*^2^ test.

The association between analyzed variables and survival was evaluated using Cox proportional hazard regression analysis. Both univariate and multivariable linear models were used. For the multivariable analysis, established predictors of poor outcome in HF were employed as covariates [i.e., age, sex, body mass index, etiology, New York Heart Association (NYHA) class, and LVEF]. Kaplan–Meier curves for cumulative survival were constructed to assess the impact of SH on the primary endpoint of cardiovascular mortality. Differences in event rates between the groups were compared with the Cox–Mantel log-rank test. A *P* value < 0.05 was considered statistically significant.

Statistical analysis was performed using the R statistical programming environment, version 3.5.

## Results

**Fig. 1:**
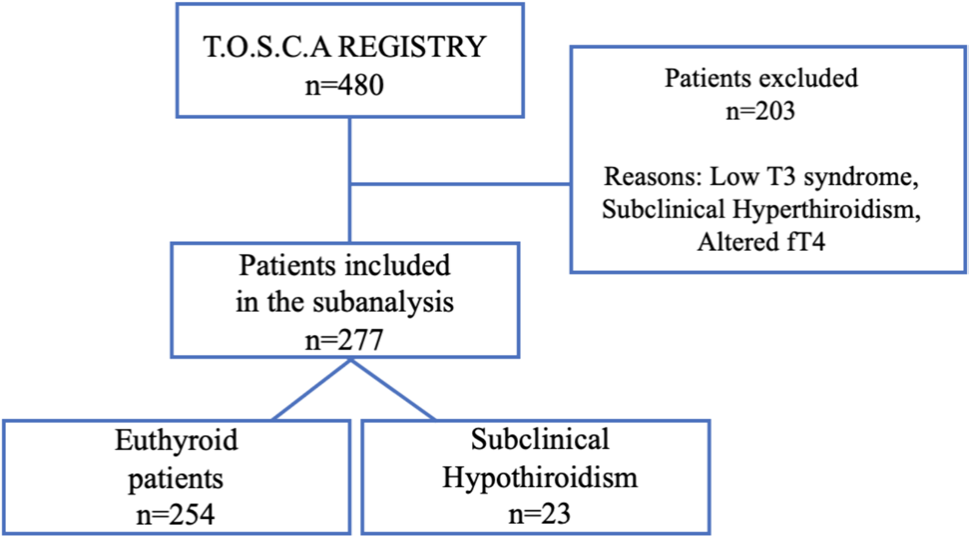
Flow-chart of the study

From the T.O.S.CA. registry cohort of 480 patients enrolled in 19 participating centers from April 2013 to July 2017 [13], 277 patients met the inclusion criteria and were included in the present analysis (Fig. 1). Of these, 254 (91.7%) patients were euthyroid and 23 (8.3%) patients had SH (87% mild vs 13% severe). No patient was lost to follow-up. Baseline demographic and clinical characteristics of the study population are described in Table 1. SH patients were older, had lower body mass index (BMI) and blood pressure, and higher NT-pro-BNP levels compared to non-SH patients. No differences were found with regard to NYHA class, HF etiology, heart rate, and ejection fraction between the two groups. As expected, SH patients showed higher TSH levels, with similar levels of fT4, compared to non-SH patients (7.7 ± 4.1 vs 1.6 ± 0.9, *p* < 0.001; 1.3 ± 0.3 vs 1.3 ± 0.3, *p* = NS, respectively).

**Table 1:** Demographic and clinical characteristics of study population

	Study population *n* = 277	Euthyroid patients *n* = 254	Subclinical hypothyroid patients *n* = 23	*p*
Age (years)	62.7 ± 11.9	62.4 ± 11.8	66 ± 12.1	0.1
Sex (M/F)	229/48	210/44	19/4	ns
NYHA				
I	30 (10.8)	28 (11)	2 (8.7)	ns
II	148 (53.4)	140 (55.1)	8 (34.7)	ns
III	88 (31.8)	75 (29.5)	13 (56.5)	ns
IV	4 (1.4)	4 (1.6)	0 (0)	ns
Etiology (% Ischemic)	150 (54.1)	137 (53.9)	13 (56.5)	ns
BMI (kg/m^2^)	29.1 ± 5.6	29.4 ± 5.7	26.7 ± 4.3	0.03
SBP (mmHg)	122 ± 17	123 ± 16	110 ± 15	< 0.001
DBP (mmHg)	75 ± 10	76 ± 10	69 ± 9	< 0.01
HR (bpm)	70 ± 12	69 ± 12	71 ± 9	ns
Left ventricular EF (%)	32.01 ± 7.4	32.2 ± 7.3	30.3 ± 7.8	ns
eGFR (mL/min per 1.73 m^2^)	90 ± 42	92 ± 42	68 ± 35	< 0.01
BNP (pg/ml)	99.3 [41.8–182.5]	94 [41–130]	156 [110–257]	0.1
NT-pro-BNP (pg/mL)	671 [226–1920]	603 [204–1829]	1640 [471–3885]	< 0.05
TSH (mlU/L)	2.1 ± 2.2	1.6 ± 0.9	7.7 ± 4.1	< 0.001
FT3 (pg/mL)	3.3 ± 0.9	3.3 ± 0.9	2.7 ± 0.8	< 0.01
FT4 (ng/dL)	1.25 ± 0.3	1.3 ± 0.3	1.3 ± 0.3	ns
Diabetes (%)	25	25	22	ns
ICD (%)	45	45	68	ns
CRT (%)	14	14	23	ns
Medication (%)				
B-blocker	88	91	91	ns
ACE-I/ARBs	54	56	50	ns
MRA	49	50	55	ns
Diuretics	75	79	83	ns
Amiodarone	19	17	50	< 0.001
Digoxin	10	11	9	ns
Antiplatelets	62	64	64	ns
Antithrombotic	34	35	32	ns
Lipid-lowering medications	63	64	68	ns

Abbreviations: *ACE-I* angiotensin-converting-enzyme inhibitors, *ARBs* angiotensin-receptor blockers, *BMI* body mass index, *HR* heart rate, *CRT* cardiac resynchronization therapy, *CRT* cardiac resynchronization therapy, *DBP* diastolic blood pressure, *ICD* implantable cardioverter defibrillator, *M/F* male/female, *NYHA* New York Heart Association, *SBP* systolic blood pressure, *TSH* thyroid-stimulating hormone

**Fig. 2:**
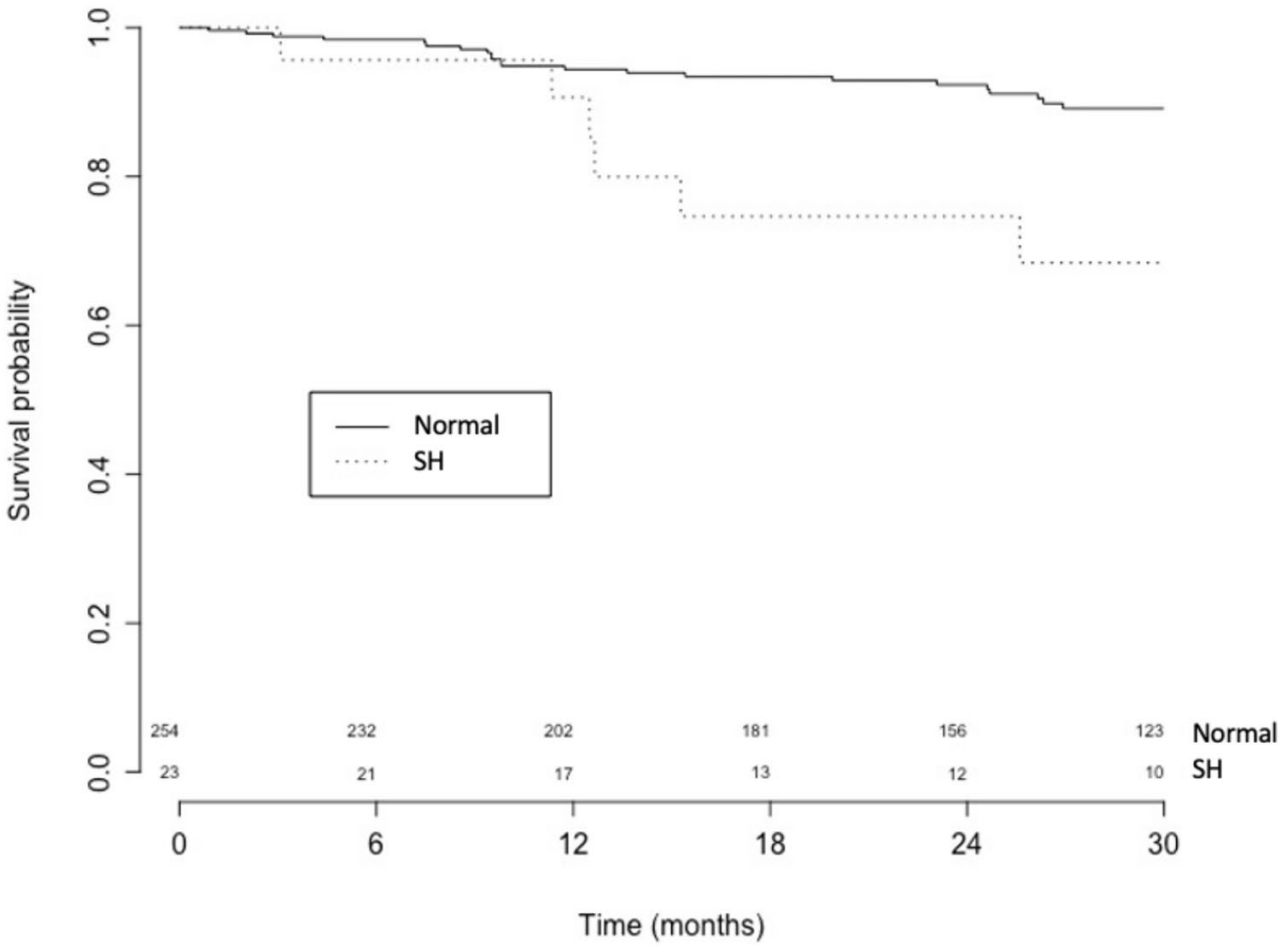
Occurrence of the primary endpoint in patients with subclinical hypothyroidism. Kaplan–Meier analysis of cardiovascular death (primary endpoint), in patients with subclinical hypothyroidism (*n* = 23) vs. euthyroid patients (*n* = 254). Log-rank testing was applied for calculation of *p*-values

At the end of the 30-month follow-up period, 131 non-SH patients (51.6%) experienced the primary endpoint of cardiovascular death compared with 13 patients (56.5%) in SH group (*p* < 0.01), as shown in Fig. 2.

### Cox regression analysis

At univariate Cox proportional hazard regression analyses, the presence of SH was associated with cardiovascular mortality (HR 3.2 [1.3–7.9], *p* = 0.01) (Table 2). After correction for established predictors of poor outcome in HF employed as covariates in a multivariable Cox regression model (i.e., age, sex, body mass index, etiology, NYHA class, and LVEF), the presence of SH resulted to be an independent predictor of outcome (HR: 2.96, [1.13–7.74], *p* = 0.03) (Table 3).

**Table 2:** Univariable model of Cox proportional hazard analysis

Variables	HR	95% CI	*p*
Subclinical hypothyroidism	3.2	1.3–7.9	0.01
Age	1.02	0.99–1.06	0.2
Sex (female)	0.4	0.1–1.5	0.2
NYHA			
II	2.6	0.34–20.34	0.3
III	5.9	0.78–45.08	0.1
IV	8.8	0.55–140.56	0.1
HF etiology (ischemic)	1.3	0.61–2.79	0.5
BMI	1	0.93–1.06	0.9

**Table 3:** Multivariable model of Cox proportional hazard analysis

Variables	HR	95% CI	*p*
Subclinical hypothyroidism	2.96	1.13–7.74	0.03
Age	1.03	0.99–1.07	0.15
Sex (female)	0.31	0.1–1.07	0.13
NYHA II	2.5	0.32–19.8	0.38
NYHA III	3.5	0.45–27.75	0.23
NYHA IV	2.1	0.11–37.72	0.62
HF etiology (ischemic)	0.97	0.43–2.18	0.94
BMI	1.07	0.99–1.16	0.08

## Discussion

In the present study, we document a relationship between SH and cardiovascular mortality in HF patients with ejection fraction ≤ 45%, which is maintained even after correction with established predictors of poor outcome in HF. The novelty of our study is that this association was observed in a population: (1) consisting mainly of patients with mild SH, (2) enrolled in a prospective registry, specifically designed to investigate the role of endocrine alterations in a large cohort of well-characterized chronic HF patients, (3) after exclusion of patients with other thyroid abnormalities (i.e., low T3 syndrome, and clinical thyroid disorders), (4) with a long-term follow-up, and (5) after adjustment for the main predictors of poor outcome in HF.

The relationship between clinical thyroid disorders and cardiovascular outcomes in patients with HF has long been evaluated, indeed, ESC HF guidelines recommend the assessment of thyroid function in all patients, as both hypo- and hyperthyroidism may cause or precipitate HF [7]. In contrast, the burden of SH on the prognosis of patients with HF is still debated and, replacement therapy is usually prescribed only for TSH levels > 10 mIU/L, particularly in patients < 70 years [14, 15].

Studies reporting outcomes as a function of SH in HF have shown inconsistent results.

In the study of Frey and colleagues, SH was diagnosed in 4.6% of HF patients but was not associated with increased mortality risk, even after adjusting for age [16]. Similarly, in the CORONA trial, hypothyroidism did not result an independent predictor of adverse clinical outcomes in HF, when NT-proBNP levels were included in multivariable models [17]. Chen and collaborators studied a large HF population dividing patients into quartiles based on TSH levels: highest TSH levels were associated with increased mortality compared with patients with lower TSH levels [6]. However, the results of these two studies were hampered by the absence of fT3 and fT4 values, thus, patients with elevated TSH might have clinical hypothyroidism. Wang et al. analyzed complete thyroid function profile in 572 consecutive hospitalized HF patients with idiopathic dilated cardiomyopathy, reporting a 23% prevalence of SH which was an independent predictor of mortality. Thyroid hormone profile was assessed before patients’ discharge, when HF symptoms were controlled with oral HF medications [18]. Hayashi et al. reported SH in 58/274 patients hospitalized for acute decompensated HF. SH was associated with a significantly lower long-term survival rate compared with patients with normal thyroid function. However, increased TSH levels were detected upon admission for acute decompensated HF, thus, the authors investigated the relationship between SH and outcomes in HF in a setting of patients with instability of the hypothalamic–pituitary–thyroid axis [19]. Similarly, Sato et al. revealed an association between SH and adverse prognosis in HF patients, associated with lower peak breath-by-breath oxygen consumption and higher mean pulmonary arterial pressure. Unlike our study, their study population was enrolled during hospitalization for decompensated HF and the presence of low T3 syndrome was not considered [20]. Kannan et al. examined the prevalence of thyroid dysfunctions and its association with cardiovascular outcomes in a large cohort of HF patients. The authors showed that SH, with a TSH levels between 7.00 and 19.99 mIU/L, was associated with increased risk of the composite end point of ventricular assist device placement, heart transplantation, or death, whereas SH with TSH levels between 4.51 and 6.99 mIU/L was not associated with outcomes. In our study, the association between SH and cardiovascular death in HF was observed in a population consisting mainly of patients with mild SH. However, in the study of Kannan et al., data on clinical characteristics, HF severity, comorbid conditions, and medications in the subgroup of SH patients, that may explain the differences with our results, were not reported [5].

Several mechanisms could be responsible for the effects of SH in HF. Impairment of left ventricular systolic and diastolic function has been described in patients with SH [20–23] and a robust MRI study demonstrated that SH was associated with decreased stroke volume and cardiac output, with a complete normalization following thyroxine replacement therapy [21]. Patients with SH have shown vascular abnormalities, involving both reduced vascular compliance and impaired endothelial function through reduction of nitric oxide availability [24, 25]. Berezin et al. suggested that SH in patients with HF might be associated with an impaired release pattern of circulating extracellular microparticles with a predominance of apoptotic-derived microparticles [26]. In patients with SH, TSH levels have been directly correlated with levels of inflammatory markers, such as CRP, interleukin-6 (IL-6), and erythrocyte sedimentation rate (ESR) [27–29]. Moreover, SH was associated with higher levels of systolic and diastolic blood pressure, total cholesterol, LDL-c, and triglycerides, all risk factors for coronary heart disease development [30, 31]. The effect of SH on renal hemodynamics remains unclear with conflicting results in the literature [32, 33]. However, in patients with kidney failure, requiring hemodialysis, SH was associated with higher mortality compared with patients with normal thyroid function [34].

The results of the present study pave the way for implementing thyroid hormone replacement therapy in HF. Current guidelines provide general recommendations to correct SH when the TSH is > 10 mIU/L, particularly in patients < 70 years, and to consider replacement therapy at lower TSH levels (7–10 mIU/L) [7, 14, 15, 35]. Indeed, no randomized trials investigated efficacy and safety of replacement therapy in HF patients with SH [8]. However, preclinical investigations and some non-randomized controlled clinical studies have shown a beneficial effects of thyroid hormone therapy on cardiac function [36–38]. In our study, we mainly enrolled patients affected by mild SH (TSH between 4 and 10 mIU/L), who should not receive replacement treatment according to current guidelines. Our results strengthen the need of prospective cardiovascular outcome studies and dose–response trials to understand better define the prognostic impact of levothyroxine replacement therapy in CHF patients.

In our previous work, a significant association between low T3 syndrome and the composite endpoint of all-cause mortality and cardiovascular hospitalization has been demonstrated in HF patients [4]. In addition, the results of the present study further highlight the importance of assessing thyroid profile in patients with HF to drive therapies and improve prognosis.

## Limitations

Our study presents several limitations. First, the observational character of our study is acknowledged [11]. However, our study population is well characterized, and we have assessed outcomes over a long follow-up period which justifies the relatively small sample size. Second, patients did not receive drugs currently recommended in the treatment of HF (i.e., ARNI and SGLT2i), since the study ended in July 2017. Finally, despite we tried to adjust for clinically relevant parameters, it was not possible to correct our results for all variables that may affect HF outcome.

## Conclusions

The results of the present study suggest that SH is associated with adverse prognosis in HF patients, with multiple implications on clinical practice. Since thyroid tests are widely available and inexpensive, TSH, fT3, and fT4 should be assessed in all HF patients to detect early clinical and subclinical disorders, to evaluate replacement therapy and to improve prognosis. Further larger studies are needed to confirm the association between SH and HF, and to evaluate the role of thyroid hormone replacement therapy, especially in patients with mild SH.

## Data Availability

Not applicable.
