# Correction: Subclinical hypothyroidism predicts outcome in heart failure: insights from the T.O.S.CA. registry

**DOI:** 10.1007/s11739-024-03717-1

**Published:** 2024-07-23

**Authors:** Mariarosaria De Luca, Roberta D’Assante, Massimo Iacoviello, Vincenzo Triggiani, Giuseppe Rengo, Alfredo De Giorgi, Giuseppe Limongelli, Daniele Masarone, Maurizio Volterrani, Antonio Mancini, Andrea Passantino, Pasquale Perrone Filardi, Angela Sciacqua, Olga Vriz, Roberto Castello, Michela Campo, Giuseppe Lisco, Pietro Amedeo Modesti, Stefania Paolillo, Toru Suzuki, Andrea Salzano, Alberto Maria Marra, Eduardo Bossone, Antonio Cittadini, T.O.S.CA. Investigators

**Affiliations:** 1 Department of Translational Medical Sciences, Federico II University, Naples, Italy; 2 Cardiology Unit, Department of Medical and Surgical Sciences, University of Foggia, 71122 Foggia, Italy; 3 Interdisciplinary Department of Medicine-Section of Internal Medicine, Geriatrics, Endocrinology and Rare Diseases, University of Bari ‘A Moro’, Bari, Italy; 4 Istituti Clinici Scientifici ICS Maugeri-S.P.A.-Istituti Di Ricovero E Cura a Carattere Scientifico (IRCCS) Istituto Scientifico Di Telese Terme, Telese, Italy; 5 Clinical Medicine Unit, Department of Medicine, Azienda Ospedaliero-Universitaria S. Anna, Ferrara, Italy; 6 Division of Cardiology, Monaldi Hospital, Azienda Ospedaliera Dei Colli, University of Campania Luigi Vanvitelli, Caserta, Italy; 7 Department of Medical Sciences, IRCCS San Raffaele Pisana, Rome, Italy; 8 Operative Unit of Endocrinology, Catholic University of the Sacred Heart, Rome, Italy; 9 Scientific Clinical Institutes Maugeri, IRCCS, Bari, Italy; 10 Department of Advanced Biomedical Sciences, Federico II University, Naples, Italy; 11 Department of Medical and Surgical Sciences, University Magna Græcia of Catanzaro, Catanzaro, Italy; 12 Heart Center Department, King Faisal Hospital & Research Center, Riyadh, Saudi Arabia; 13 Division of General Medicine, Azienda Ospedaliera Universitaria Integrata, Verona, Italy; 14 Department of Medical and Surgical Sciences, Unit of Endocrinology and Metabolic Diseases, University of Foggia, Foggia, Italy; 15 Department of Medical Sciences, School of Medicine, Pharmacy and Prevention, University of Ferrara, Ferrara, Italy; 16 Department of Cardiovascular Sciences, University of Leicester, NIHR Biomedical Research Centre, Glenfield Hospital, Leicester, UK; 17 Cardiology Unit, A.O.R.N. Antonio Cardarelli, Naples, Italy; 18 Department of Public Health, University “Federico II” of Naples, Naples, Italy; 19 Division of Internal Medicine & Metabolism & Rehabilitation, University Federico II, 80131 Naples, Italy

**Correction: Internal and Emergency Medicine** 10.1007/s11739-024-03665-w

In this article the funding section was incorrectly written as ‘Open access funding provided by Università degli Studi di Napoli Federico II within the CRUI-CARE Agreement’ it should have been ‘Open access funding provided by Università degli Studi di Napoli Federico II within the CRUI-CARE Agreement. This work was partially supported by funding of the Italian Ministry of Health [Ricerca corrente]’.

The original article has been corrected.

